# On the Very-Long-Term Effect of Managing One’s Own Memory: The Intention to Forget Improves Recognition After a Year’s Delay

**DOI:** 10.5964/ejop.v14i4.1606

**Published:** 2018-11-30

**Authors:** Veronika V. Nourkova, Alena A. Gofman, Mikhail D. Kozlov

**Affiliations:** a Department of General Psychology, Lomonosov Moscow State University, Moscow, Russia; b Luisenklinik, Department of Psychology, Bad Duerrheim, Germany; Department of Psychology, Webster University Geneva, Geneva, Switzerland; University of Bari "A. Moro", Bari, Italy

**Keywords:** intentionality, intentional forgetting, mnemonic goal, mnemonic strategies, free recall, recognition

## Abstract

While such factors as demand characteristics, encoding, and retrieval inhibition were shown to be significant in producing the directed forgetting effect, no attention was paid to whether the intention to manage one’s own memory, per se, matters. In the present article, we addressed this important gap in the literature. To control the quality of encoding we ensured that both the to-be-remembered (TBR) and to-be-forgotten (TBF) items were genuinely learned before the manipulation. We used extremely long delays between the memory instructions and testing to release inhibition associated with the content of instructions. 98 participants demonstrated flawless recall of 12 Russian - made up language word pairs. They then viewed each Russian word from a pair once, with randomized instructions “Forget”, “Remember”, “Repeat”, or a short cognitive task. Self-reports on the mnemonic strategies were collected. Free recall and recognition tests were administered three times - 45 minutes, a month and a year (N = 58) later. Despite a strong incentive to recall all word pairs, fewer TBF pairs were recalled in comparison with TBR pairs, both after 45 minutes and after one month’s delay. Recognition among all conditions was equally high. A year later free recall was close to zero. In contrast, the TBR and TBF pairs were recognized equally better than pairs presented in “Repeat” and “Task” conditions. Thus, our results show that the intention to manage one’s own memory enhances the accessibility of memories at a very long time delay, no matter what type of instruction is issued.

People often make efforts to forget incorrect information. Are they really able to succeed in doing so? Does the intention to forget decrease the probability to retrieve the wrong information later on, or is it that the harder one tries to forget something the easier it pops into mind (see Wenzlaff & Wegner, 2000, for review)? Is it possible that the very intention to manage one’s own memory stands behind the striking inability to ban the unwanted memories? To address these questions, we first survey the main findings relevant to the problem of voluntary forgetting and then report our empirical study, devised to shed light on the role of mnemonic goals in memory accessibility.

The fact that participants show poor memory performance when instructed to forget in a lab setting is known as directed forgetting (Basden, Basden, & Gargano, 1993; Bjork, LaBerge, & Legrand, 1968; MacLeod, 1998). Typically, dozens of items are presented at a rapid rate followed by the instruction to forget (to-be-forgotten items - TBF) or to remember (to-be-remembered items - TBR). The effectiveness of directed forgetting has been documented by various kinds of tests: free recall, cued recall, recognition (Benjamin, 2006), word completion task (MacLeod, 1989; McKinney & Woodward, 2004; Paller, 1990), lexical decision task (Fleck et al., 2001), priming (Marks & Dulaney, 2001), remember-know judgments (Gardiner, Gawlik, & Richardson-Klavehn, 1994), reaction time of visual detection (Zacks, Radvansky, & Hasher, 1996), and eye-tracking (Thompson & Taylor, 2015). There is an inverse relationship between the sensitivity of the final memory test and the magnitude of the effect, in such a way that the more indirect the test, the smaller the effect (Basden et al., 1993). Although the effect of directed forgetting appears to be temporary and vanishes after a period not exceeding one week (Nørby, Lange, & Larsen, 2010; Wheeler, 1995), it has received extensive attention due to both practical and theoretical reasons, opening an avenue to understanding the basic processes of human memory. The general aims of the present study were:

1. To replicate the basic directed forgetting effect using a small set of genuinely learned items after realistically long delays between the memory instructions and testing, and both direct (free recall) and indirect (recognition) tests.
2. To examine whether a mnemonic goal (like the intention to remember OR to forget), might actually yield better memory in comparison with control conditions.

## Mechanisms Underlying the Directed Forgetting Effect

The most trivial explanation of the directed forgetting effect is the good participant hypothesis (Nichols & Maner, 2008). On this view, participants tend to interpret the instruction to forget as a demand to not report the TBF items on the subsequent recall test. In such a case, worse recall of the TBF items would not necessarily have anything to do with forgetting. Instead, it attests the excellent memories for study-phase instructions. However, MacLeod (1999) demonstrated that demand characteristics cannot fully account for the deficit in reproducing the TBF items. Participants did not reproduce significantly more TBF items after they were told that they would be paid for each previously unreported item.

Further, relatively poor recall and recognition observed after the instruction to forget may indicate a lack of encoding. Since the typical research procedure employs novel material at the learning phase, it seems likely that upon receiving the forget and remember instructions participants simply cease rehearsal of the TBF items and enhance rehearsal of the TBR items. Thus, the directed forgetting effect might merely reflect better memorizing of the repeatedly rehearsed TBR items rather than forgetting of the TBF items (Bjork, 1972; MacLeod, 1998; Taylor, Cutmore, & Pries, 2018). Accordingly, recognition increases for both the TBR and TBF items as a function of time available for rehearsal before any instruction (either to remember or to forget) is issued (Woodward, Bjork, & Jongeward, 1973). Similarly, it has recently been shown that the prevention of rehearsal by articulatory suppression reduces the directed forgetting effect at recognition probes, leveling the differences between the TBR and TBF items (Festini & Reuter-Lorenz, 2017). More interestingly, the prolongation of a blank time interval after the specific instruction, when the rehearsal of the TBF items is assumed to be terminated, actually provoked higher recognition for both the TBR and TBF items (Lee, Lee, & Tsai, 2007). The latter result allows us to speculate that rehearsal continues automatically in the absence of a competing stimulus (Lee & Lee, 2011).

However, the maintenance of rehearsal is not the only process engaged during encoding. To our knowledge, only one study has attempted to control the quality of encoding as an outcome of level-of-processing manipulations (Dulaney, Marks, & Link, 2004). Three conditions addressing different levels of processing were employed. In the first condition, participants counted the number of vowels in the words by pressing the appropriate number (shallow level), in the second, participants read the words and then pressed one of three response keys (medium level), and in the third, they read the words and then pressed a key corresponding to their subjective rating of the word’s pleasantness (deep level). Words were then randomly assigned to the TBR or TBF conditions. The results revealed that the deep processing (pleasantness) led to better recall and recognition for both the TBR and TBF items.

It is worth noting that in all studies mentioned above the effect of encoding was clearly pronounced at recognition tests, only. In free recall the data were quite mixed. Thus, the directed forgetting effect can only partly be explained in terms of encoding quality; another mechanism, related to consciousness and working memory functioning, seems to be in play. Anderson, for instance, posits that directed forgetting is produced by inhibitory processes acting over the TBF items. According to this powerful account, fulfillment of the instruction to forget is achieved by an active, cognitively effortful process of retrieval inhibition (for reviews, see Anderson & Hanslmayr, 2014; Anderson & Huddleston, 2012).

Paradoxically, similar to the demand characteristic account, inhibition control theory implies that a person remembers the unwanted stimulus extremely well, being able to keep it out of retrieval even if a reminder is present. In Anderson’s “think / no-think” paradigm participants are trained to respond to a reminder word paired with a target word. Next, they are asked to retrieve target words associated with half of the reminders and to avoid retrieving in response to the other half. At the final memory test, the standard directed forgetting effect appears: People exhibit better memory for words that they were instructed to retrieve compared to words that they were instructed to suppress (Anderson & Green, 2001; Anderson et al., 2004). There are at least four research paradigms that provide substantial empirical support to inhibition control theory.

First, there is a large number of findings showing that memories that are thought to be forgotten, may be retrieved later. The spontaneous recovery of the previously intentionally forgotten material was demonstrated employing both the so called list method and the so called item method. In the former method, two lists are learned, and afterwards one is designated as to-be-forgotten (Wheeler, 1995). In the latter method, items are presented one at a time, each followed by an instruction to remember or forget (Geiselman & Bagheri, 1985). Secondly, according to the executive deficit hypothesis (Levy & Anderson, 2008), people with an impaired executive control ability (due to cognitive aging or various mental diseases) reported to experience difficulties with inhibiting unwanted memories (Anderson, Reinholz, Kuhl, & Mayr, 2011; Gardiner et al., 1994). In this vein, it would be indicative if the reversible release from inhibition appears after sleep deprivation or alcohol consumption. Third, there is the cognitive load hypothesis, even though the authors consider this hypothesis as independent or even partly competing (Lee, 2012). According to this hypothesis, a cognitive resource is employed for active forgetting. If so, the more resources are assigned to the TBR items processing, the fewer resources are assigned to the forgetting of the TBF items. Hence, TBF items would be recalled better. Lee’s results were consistent with this prediction: Participants recalled fewer TBF items when TBR items had to be just named (low resource consuming condition), than when receiving a direct instruction to remember TBR items (high resource consuming condition). In other words, the cognitive resource is required to keep the target items forgotten. Quite similarly, participants performing focused breathing techniques as a secondary task during the item-method directed forgetting later recognized the TBF items better than controls (Gamboa, Garcia-Campayo, Müller, & von Wegner, 2017). Finally, one of the most important pieces of evidence for an active inhibitory framework behind the intentional forgetting phenomena is that this process operates strategically. Analyzing self-reports, collected after the directed forgetting trials, Levy and Anderson (2008) discerned 16 forgetting strategies, for instance, generating a masking mental image or thinking of an alternative word. Although, as the authors noted, there are various ways to achieve successful suppression of unwanted memories, they may be classified as relying upon either substitution, that is, occupying the limited focus of awareness with another material, or upon direct suppression (Benoit & Anderson, 2012). Indeed, different brain regions associated with those two mechanisms were identified. During substitution interactions between left caudal and midventrolateral prefrontal cortex were detected, while systemic inhibition of hippocampal processing that originates from the right dorsolateral prefrontal cortex was observed during direct suppression (Benoit & Anderson, 2012). Moreover, results from Foster and Sahakyan (2011) suggested that significant directed forgetting was detected only among those participants who reported engaging in forgetting strategies, for example, stopping rehearsal or starting to think about unrelated things. Accordingly, elderly participants in Murray et al.’s studies were able to suppress target memories only after being explicitly instructed in strategies to keep the TBF words out of mind (Murray, Anderson, & Kensinger, 2015; Murray, Muscatell, & Kensinger, 2011). However, it seems that there is still much to learn about the variety of strategies people might employ to inhibit “forbidden” memories especially in respect to their effectiveness.

### The Concern of Intentionality

Taking additionally into account that memory traces may decay with time or because of other reasons (for discussion see, Hardt, Nader, & Nadel, 2013; Roediger, Weinstein, & Agarwal, 2010), it appears that forgetting is a multi-factorial process. Such factors as demand characteristics, encoding, processing, and retrieval inhibition proved to play significant roles in producing the directed forgetting effect. However, there are important themes that often go unappreciated. Ironically talking about the intentional action of forgetting, psychologists often pay little attention to intentionality. For instance, retrieval inhibition in directed forgetting is viewed as identical to automatic retrieval-induced forgetting, which, however, occurs without a goal to forget (Storm et al., 2015). To date, there has not been any systematic research isolating a conscious mnemonic goal from other variables thought to affect forgetting.

Is it necessary to remember the initial intention to forget to keep the item forgotten or inaccessible? Some evidence suggests that it is. For instance, it has been demonstrated that the more attempts to confront reminders to unwanted memories participants made, the more they succeed in suppressing recall (Anderson & Huddleston, 2012). This result implies that learning not to recall specific items constitutes an automation of voluntary inhibition. In contrast, Taylor et al.’s (2018) data show that the magnitude of the directed forgetting effect was not contingent upon whether participants were informed about the initial conditions that the items were presented in.

Yet another possibility is that any intentions to manage one’s own memory induces a specific mode of processing, improving the strength of memory traces. If the goal is to remember, it facilitates retrieval, while if the goal is to forget it inhibits retrieval. Thus, assuming possible differences in encoding are controlled for, both the TBR and TBF items should be equally accessible in recognition tests after a long delay, when the initial instructions no longer matter. In contrast, we would observe non-directed forgetting of control items not involved in voluntary processing. In support of this prediction, Gao et al. (2016) showed lower recognition accuracy for items presented in the control condition, in which remember/forget instructions were not given to participants, compared to both the TBR and TBF items. However, given that the interval between the study phase and test phase was only 5 minutes, the ecological validity of that study is questionable (an issue that incidentally applies to the majority of studies on directed forgetting).

### The Present Research

The main aim of the present research was to examine the hypothesis that a conscious mental action, performed with the intention to manage one’s own memory, leads to improvement of memory. We argue that this hypothesis is best tested at long delays between experimental manipulation and retrieval, when people stop being aware of the specifics of their previous mnemonic intentions (to remember or to forget). Because of that reason, free recall and recognition tests were administered three times - 45 minutes, a month, and a year after the experimental manipulation. Since we assumed that the mnemonic goal makes an independent impact on the subsequent accessibility of the material, we intended to isolate as many extraneous variables in the design as possible. First, to avoid concerns of encoding differences across conditions we ensured that all items were genuinely memorized before the main experimental manipulation. Indeed, we maintain that only material that has been securely encoded in the first place can be considered to be forgotten later on. In this strict interpretation of forgetting we followed Tulving’s (1974) definition of forgetting as “the inability to recall something now that could be recalled on an earlier occasion” (Tulving, 1974, p. 74). Second, to reduce the possibility of prior associations affecting memorability of the TBR and TBF material, we used made-up words consisting of two syllables, each. Third, to disentangle the natural conjunction of a goal and the means that are used to achieve that goal, we modified the typical item-method directed forgetting paradigm by adding a “repetition without a mnemonic goal” condition. The condition “repetition” was included in the experimental design because rehearsal was demonstrated to be the most common strategy for intentional remembering. We assumed that the difference in performance between the typical TBR condition (mnemonic goal + repetition) and the “repetition without a mnemonic goal” condition would discern the role of the intention.

## Method

### Participants

One hundred and sixty-six students enrolled in a psychology course conducted by one of the authors (VN) at the Lomonosov Moscow State University, were invited to participate. As part of a course assignment, the students were provided with a printed list of 12-word pairs, where a Russian (native language) word was always paired with a corresponding made up “foreign language” word. The students were asked to learn the word pairs over the next two weeks. Of the 166 students, 98 (aged 19-22, 83% females) succeeded in flawlessly recalling all 12-word pairs in a free recall test administered two weeks later, requiring the recall of each Russian word with its corresponding “translation” and vice versa. These 98 then took part in the main experimental manipulation. All of them were tested after 45 minutes and one month after the experimental procedure described below. A year later 58 of them volunteered to participate in a re-test.

### Materials

An item-cued directed forgetting paradigm was used in the study. For the experimental manipulation an MS PowerPoint presentation featuring the 12 Russian words, one word per slide, was used. At the beginning of the presentation, detailed explanations of the experimental instructions were provided. The participants were informed that the “Remember” instruction meant that the corresponding made up translation of the featured word was to be retained in memory. Seeing the “Forget” instruction meant that the corresponding made-up word was to be forgotten. The “Repeat” instruction meant that the Russian word featured on the slide was to be repeated out loud 10 times, in unison with the experimenter who set the pace. Finally, if a slide featured a cognitive task, the participants were to solve it and write down the answer on a blank sheet of paper in front of them. These explanations were followed by a practice block to ensure that the participants knew what each experimental instruction required of them. Each slide was presented, with the instruction underneath the Russian word, for 10 seconds. Each word was randomly assigned to the instruction “Remember”, “Forget”, “Repeat”, or a short cognitive task (e.g. “solve: (15-5)*2=…”). To account for word-item based confounds, each quarter of the sample presentation was altered in the way that the word pairs were counterbalanced with respect to conditions.

### Design

The study was a within-subjects repeated measures design with two independent variables. The first independent variable was the condition at manipulation (Remember, Forget, Repeat and Task), the second independent variable was the time interval between manipulation and test. The dependent variable was the mean number of word pairs that were freely recalled and recognized per participant. A free recall test was administered 45 minutes, a month and a year after the main manipulation. A recognition test was administered a month and a year after the main manipulation (recognition after 45 minutes was assumed to be at ceiling).

### Procedure

The 98 students who had demonstrated flawless recall of the 12-word pairs in individual examination attended the 10 minutes long PowerPoint presentation containing the experimental manipulation, separately in four groups (23/24/25/25) at the beginning of their classes on the same day. The PowerPoint presentation was given only once. After the classes, that is, 45 minutes later, participants were encouraged to recall and write down all of the initially learned 12 Russian words with the corresponding translations. Cheating was forbidden; adherence to that rule was monitored by 3 research assistants and the participants were seated in the classroom so that each participant occupied a separate desk. To exclude not reporting a TBF item due to demand characteristics, participants were told that the most successful quartile of participants would be announced and released from filling in a lengthy questionnaire specially constructed for participants with poor memory abilities. In fact, they were all told that they had succeeded. Self-reports on the methods, which participants used to accomplish the instructions were collected after the first test. One month and a year after the experimental manipulation two further free recall tests were administered. After these free recall tests, participants also performed recognition tests wherein they had to recognize the 12 made up words among 24 lures. These tests took about 15 minutes to complete. For a one year follow-up, the participants were contacted via departmental e-mail and those who responded were tested individually. At the final test session, after completing the recognition procedure, participants were presented a list with all the initially learned 12-word pairs and were asked to mark the TBR pairs as “R” and the TBF pairs as “F”.

## Results

Only those participants who took part in all three tests (*n* = 58) were included in the main calculations. Only word pairs where the appropriate, and correctly spelled, “translation” of a Russian word had been written down at free recall were counted as correct. However, all data collected on self-reported strategies of remembering and forgetting (*n* = 98) was analyzed.

### Recall

A 3 (Time: 45 minutes, 1 month, 1 year) X 4 (Instruction: Remember, Forget, Repeat, Task) mixed ANOVA, revealed a significant effect of Time, *F*(2, 56) = 423.183, *p* < .001, MSE = .751, η^2^_p_ = 0.881, a significant effect of Instruction, *F*(3, 55) = 12.241, *p* < .001, MSE = .386, η^2^_p_ = 0.177, and a reliable “Time x Instruction” interaction, *F*(6, 52) = 4.034, *p* = .001, MSE = .318, η^2^_p_ = 0.066. To clarify the differences between tests within each condition and between conditions at each time point we employed Bonferroni-corrected paired-sample *t*-tests for dependent samples (*p* < .008 for 6 comparisons), and Cohen’s weighted d effect sizes were estimated (Cohen, 1988).

**Figure 1:**
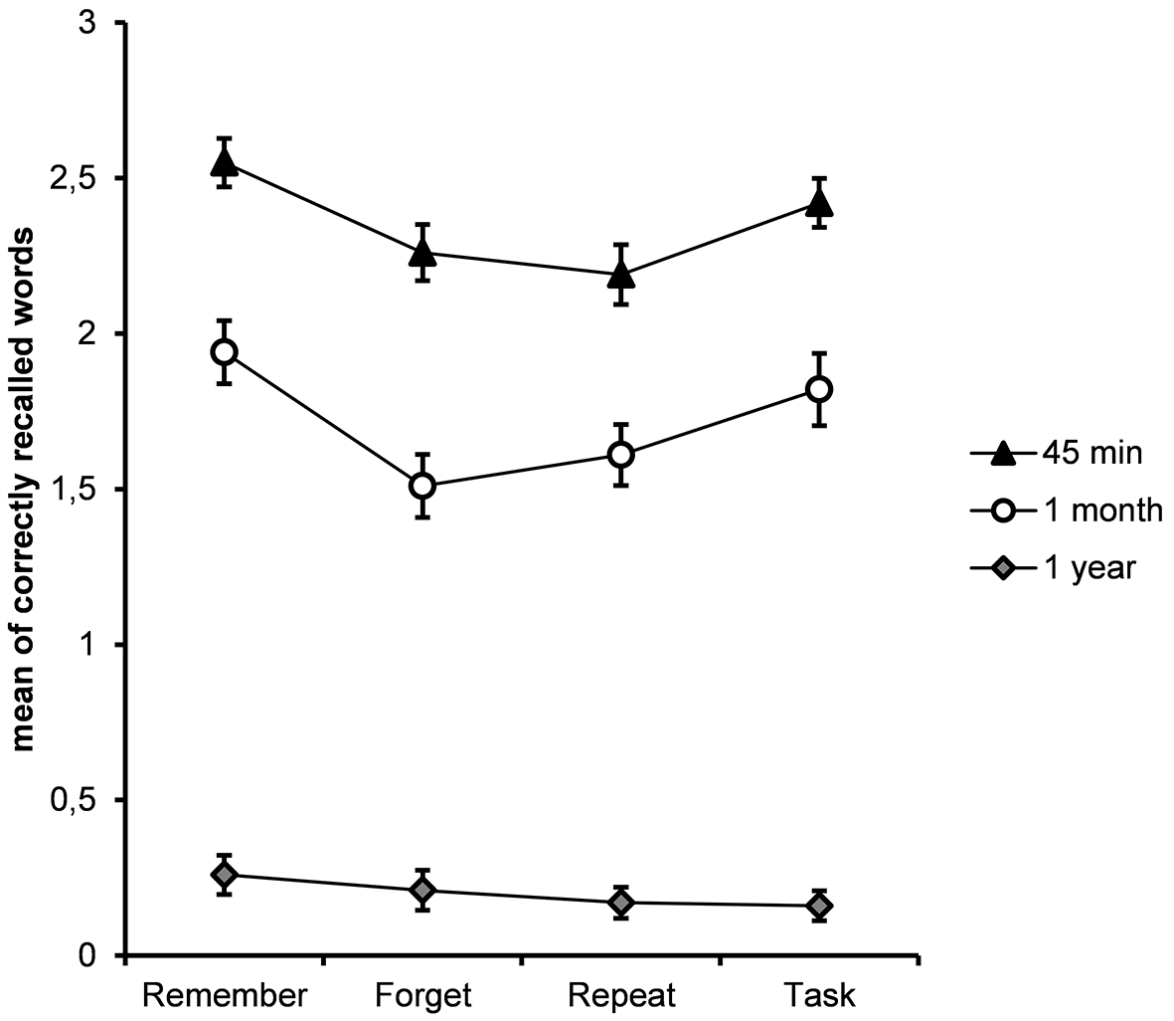
Mean of correctly recalled word pairs in each condition on recall tests administered 45min, 1 month and 1 year after the manipulation. The error bars correspond to one standard error.

Not surprisingly, free recall significantly decreased from time 1 to time 2 and from time 2 to time 3, and this was true across all instructions at *p* < .001 (see Figure 1).

Nevertheless, the standard directed forgetting effect was observed at free recall Tests 1 and 2. While word pairs presented during manipulation in the “Remember” and “Task” conditions did not differ from each other both at Test 1 (*t*(57) = 1.211, *p* = .231) and at Test 2 (*t*(57) = 0.843, *p* = .403), the TBF items were significantly inhibited in comparison to the TBR items (for Test 1 *t*(57) = 3.160, *p* = .003, Cohen’s *d* = 0.511; for Test 2 *t*(57) = 3.563, *p* = .001, Cohen’s *d* = 0.496). The differences between the TBF condition and the “Task” condition reached significance at Test 2 only *t*(57) = 3.953, *p* < .001, Cohen’s *d* = 0.574).

The “Repeat” condition failed in producing a recall benefit relative to the TBR condition at both tests (for Test 1 *t*(57) = 3.657, *p* = .001, Cohen’s *d* = 0.479; for Test 2 *t*(57) = 3.947, *p* < .001, Cohen’s *d* = 0.557). Rather, free recall performance after rehearsal of the Russian words without the intention to keep remembering was equal to the TBF condition (for Test 1 *t*(57) = 0.864, *p* = .391; for Test 2 *t*(57) = 0.127, *p* = .899).

At test 3, a year later, free recall in all conditions was extremely poor with no differences between them (*F*(3, 55) = 0.892, *p* = .447, MSE = 0.135, η^2^_p_ = .015).

The main findings are described in Figure 1, which depicts the mean free recall for each condition on the three tests.

### Recognition

We did not employ recognition tests at the first memory examination (45 min.) expecting performance to be at ceiling.

A 2 (Time: 1 month, 1 year) × 4 (Instruction) mixed ANOVA showed a significant effect of Time *F*(1, 57) = 86.703, MSE = .906, *p* < .001, η^2^_p_ = 0.43, a significant effect of Instruction *F*(3, 57) = 4.396, MSE = .294, *p* = .005, η^2^_p_ = 0.037, and a reliable “Time x Instruction” interactions, *F*(3, 55) = 4.479, MSE = .324, *p* = .004, η^2^_p_ = 0.037.

**Figure 2:**
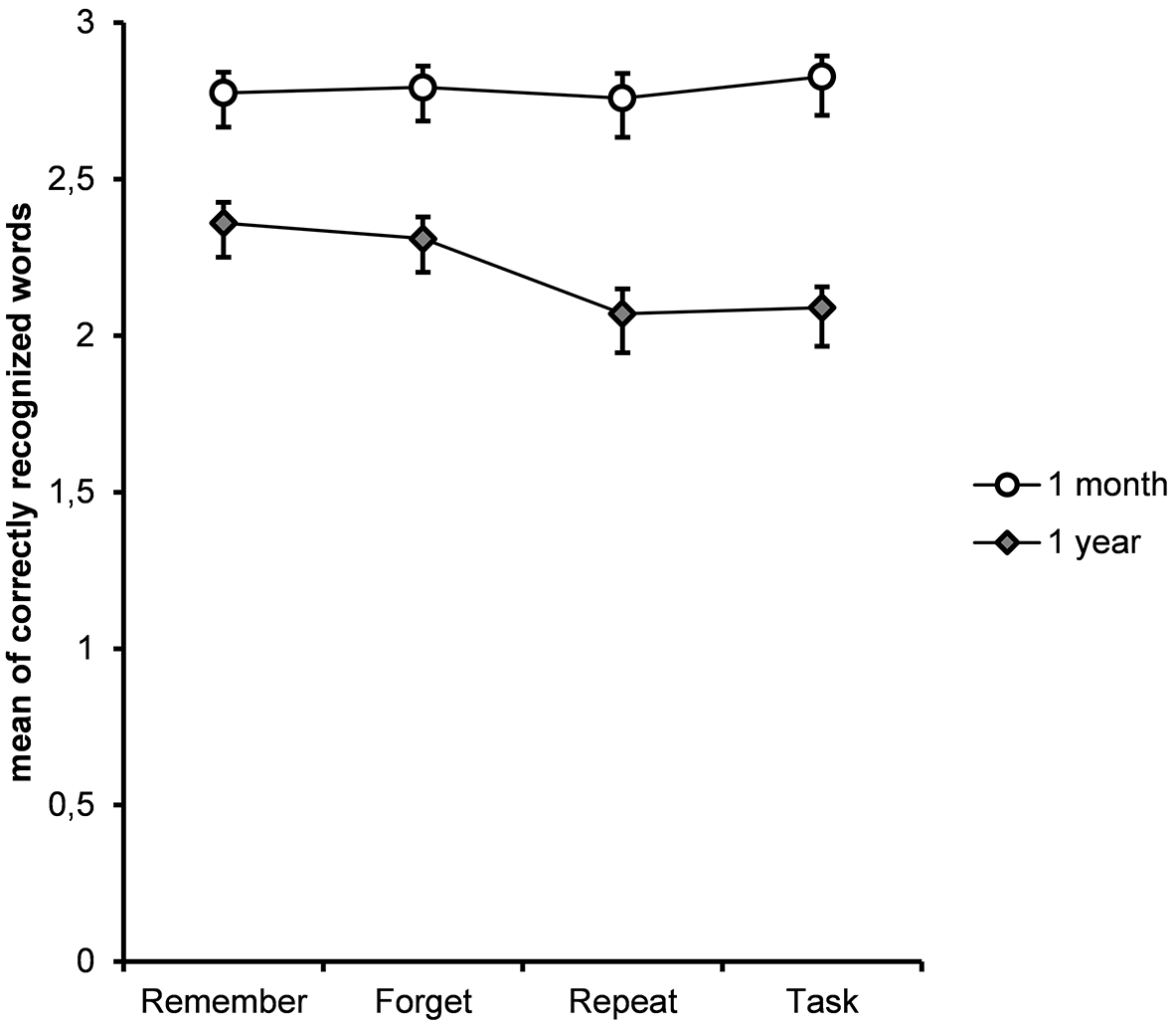
Mean of correctly recognized word pairs in each condition on the recognition tests administered 1 month and 1 year after the manipulation. The error bars correspond to one standard error.

After one month, all participants showed a high and statistically indistinguishable level of recognition of the 12 target made-up words in array of 24 lures (*F*(3, 55) = .277, *p* = .842, MSE = .182, η^2^_p_ = .005). In contrast, the results of the recognition test administered after a one year detected a specific positive effect for both conditions, which involved a mnemonic goal. Inspecting Figure 2, it is quite clear that more made-up words were recognized accurately in both the TBR and the TBF conditions than in the “Repeat” and the “Task” conditions (*F*(3, 55) = 2.992, *p* = .032, MSE = 0.442, η^2^_p_ = 0.05). A *t*-test corroborated this observation, showing that significantly more items were correctly recognized in the TBR condition (μ = 2.362, *SD* = 0.828) than in the “Repeat” condition (μ = 2.07, *SD* = 0.953), *t*(57) = 3.143, *p* = .002, Cohen’s *d* = 0.464, or in the “Task” condition (μ = 2.086, *SD* = 0.938), *t*(57) = 2.969, *p* = .004, Cohen’s *d* = 0.44. Accordingly, the recognition in the TBF condition (μ = 2.31, *SD* = 0.817) was higher than in the “Repeat” condition, *t*(57) = 2.718, *p* < .009, Cohen’s *d* = 0.384, and marginally higher than in the “Task” condition, *t*(57) = 2.402, *p* < .02, Cohen’s *d* = 0.359. At the same time, differences between the TBR and the TBF conditions and between the “Repeat” and “Task” conditions were not observed, *t*(57) = 0.645, *p* < .53, and *t*(57) = 0.245, *p* = .807, respectively.

Taken together, these findings suggest that over the course of one year, the level of recognition prevails for the mnemonic goal-driven conditions, regardless of the exact content of the goal (to forget or to remember).

### Instructions and Memory Strategies

Forty-five minutes after the main manipulation and immediately after the first memory tests, 98 participants were asked to indicate how they attempted to comply with instructions to keep remembering or to forget the word pairs. The majority of participants (70 / 71%) reported that they were rehearsing the pair during exposure to the “Remember” instruction. 17 participants (18%) declared that they were trying to establish phonological or visual associations to the made-up counterparts of the Russian words. The rest of the sample (11 / 11%) described unique methods of memorizing (e.g. “drew a mental picture”, “visualized an image of the typed word”, “stared at the screen”, “imagined oneself photographing the word pair”, “tried to make a connection between the neighboring student’s face and the word”, “ordered oneself to remember”, “wrote down the made-up word”, “allowed own brain to do whatever it wanted to do”, “let the right word come into mind”, “knocked my fingers to activate remembering”, “articulated the word very intensively”). While repetition seemed to be the most popular action for remembering, there was no comparable common strategy for forgetting. About one-third of participants (34 / 35%) reported that they switched their attention toward irrelevant thoughts or images when the instruction to forget appeared on the screen. 23 participants (23%) portrayed their forgetting action as a kind of direct mental effort to not remember. 20 participants (20%) stated that after noticing the instruction to forget, they continued to rehearse the word pair from the previous “Remember” condition. 10 participants (10%) preferred not to look at the screen at all after the “Forget” instruction was received. The rest of the sample (11 / 12%) tried to fulfill the instruction by performing unique techniques such as: “counted in silence”, “pretended to be surprised by the word on the screen”, “imagined the word written on the sheet of paper then crumpled it and threw it away”, “nominated wrong words to damage association”, “tried to fill my consciousness with a big FORGET word”, “deconstructed the word on the screen into isolated letters to lose the meaning”, “imagined the screen on fire”, “clenched my fists repeatedly”, “moved eyes from left to right and vice versa”, “imagined oneself washing memory”, “been waiting for another instruction”, “convinced oneself not to remember”. In contrast to previous data obtained by Foster and Sahakyan (2011), nobody reported doing nothing in response to the instructions.

However, we did not detect any privileged strategies for later recall neither for the “Remember” condition (χ^2^ = 6.887, *p* = .332) nor for the “Forget” condition (χ^2^ = 7.054, *p* = .854).

### The Recollection of the Initial Instruction

A year after the experimental manipulation, after completing the free recall and recognition tests, participants were provided with a printed list of the study material. No correlations were found between accuracy of recognition and accuracy of the recollection of the initial instruction either for TBR items (*r* = .142, *p* = .068) or for TBF items (*r* = .098, *p* = .464). Moreover, it looked like generally, participants classified the word pairs as TBR or TBF by chance since the ratio of correct attribution was 0.63 for the TBR items and 0.56 for the TBF items.

## Discussion

The methodology of the present research differed from a typical directed forgetting paradigm in three important ways. First, the small set of experimental items was fully learned ahead of the manipulation. Second, we conducted memory tests at very long time delays (a month and a year after the manipulation). Third, an additional condition of rehearsal without the explicit goal to remember or to forget was included in the procedure.

In the first and second free recall tests, we replicated a typical intentional forgetting effect, with genuinely learned material. Due to the fact that this effect was detected at free recall only, despite a strong incentive to recall all the learned word pairs, we attribute the result to the aftereffects of the action of inhibition. The ceiling level of recognition one month after the experimental manipulation provides additional support for this view.

Quite surprisingly, the number of recalled items in the “Repeat” condition was similar to the number of items recalled in the “Forget” condition, thus, in a way producing the standard directed forgetting effect. In our opinion, there are at least two possible explanations for this result. The first refers to research on extinction, suggesting that the associative link between two stimuli can be weakened if one stimulus is repeatedly presented in absence of the other (Schiller et al., 2010; Xue et al., 2012). This would mean that a sequence of intense repetition of the native language words, in absence of retrieval of the made-up words, could weaken or sever the link to the associated made-up words. Another explanation comes from Alfonse Jost’s (1897) law of forgetting. He posited that if two memories have the same strength but different ages, the older will benefit more from repetition than the younger one and the younger one will lose strength more rapidly than the older one. If so, we can speculate that the repetition of the native word could enhance old associations and weaken fresh ones (with made-up words).

It is further curious that recall performance in the “Task” condition was rather similar to recall performance in the “Remember” condition. This outcome makes somewhat sense though, if one bears in mind, that the item pairs had been genuinely memorized ahead of the manipulation. Thus, whether participants were told to actively keep remembering what they already knew, or were simply told to do a cognitive task should not have, and has in fact not, impacted recall performance. It did, however, impact recognition performance after a year’s time; a result that is in line with our “no-mnemonic goal — no mnemonic effect” reasoning.

Certainly, the most striking results of the study we obtained at the one-year follow up. Not surprisingly, after 12 months free recall was just slightly above zero, the same for all conditions. This leads us to believe that the aforementioned inhibition process was released, opening up a possibility to examine the state of memory traces by recognition. Indeed, participants were hardly able at this point to indicate the instructions accompanying items at the experimental manipulation. Accordingly, participants generally failed in indicating the instructions accompanying items at the experimental manipulation. In contrast to free recall, after a year, the TBR and TBF words were recognized significantly better (78.7% and 77.0% respectively) than words that were not processed in the context of any conscious mnemonic goals (69.0% for the to-be-repeated items and 69.5% for control “Task” condition). This result brings us to the conclusion that the intention to achieve any goal with mnemonic content itself protects material from forgetting at long time intervals.

It should be noted, that in our study there were overall five occasions when participants had to retrieve the originally learned material. The first free recall took place after 45 minutes, then after a month and finally after a year both recall and recognition was performed. We have to concede therefore, that the results could have been affected by the so called testing effect, that is, the improvement of memory performance after retrieval trials (Roediger & Karpicke, 2006; Roediger & Nestojko, 2015; Wheeler, Ewers, & Buonanno, 2003). The authors report that this outcome was observed on both explicit and implicit memory tests (Roediger, Gallo, & Geraci, 2002). Roediger et al. (2002) have argued that testing operates by the elaboration of existing memory traces and by strengthening and multiplying the number of ‘‘retrieval routes’’ to stored items. This suggests that even if our results had been affected by the testing effect, the effect should have uniformly acted upon participants’ performance; the observed differences between conditions can hardly be attributed to it.

To accommodate our novel results on the independent impact of mnemonic goals on delayed memory performance we look towards reconsolidation theory (McKenzie & Eichenbaum, 2011; Sara, 2000). This theory stresses that encoding (consolidation) is not a unique event. Instead, memory traces are labile when in an active state. During reconsolidation, memories can be enhanced, impaired, or updated with new information. Agren’s review (Agren, 2014) indicates that the most often used reactivation method in declarative memory reconsolidation studies is retrieval. On the other hand, not every retrieval attempt necessarily entails a reconsolidation. It only does so in situations that favor new encoding, that is, a mismatch between expected and actual contexts (Coccoz, Sandoval, Stehberg, & Delorenzi, 2013). Therefore, it is possible to interpret mnemonic goals, aimed at previously consolidated memory traces, as unexpected cues producing a reconsolidation strengthening effect. It is also worth noting that memories that cannot be consciously retrieved still can be the target of a reconsolidation process (Coccoz, Maldonado, & Delorenzi, 2011).

### Conclusion

To sum up, in the present study we observed two, possibly competing, processes. The first one was memory inhibition. The second one was memory enhancement through a goal-driven elaboration. Our results suggest that the instruction to forget inhibited respective memories at two free recall tests, but in fact, it enhanced the memory traces after a year’s delay. By that time retrieval was released from inhibitory control mechanisms, so both the TBR and TBF pairs were recognized significantly better than the other items. In our opinion, this extra-long-time delay enabled us to make out the effects of goal-driven elaboration without the competing influence of voluntary inhibition.
